# Supplementary material for: Specific quantification of inducible HIV-1 reservoir by RT-LAMP
Source: Commun Med (Lond). 2024 Jun 25;4:123. doi: 10.1038/s43856-024-00553-4 (PMC11199587; doi:10.1038/s43856-024-00553-4)
Supplement: Supplementary file 2 — Supplementary Information [file 43856_2024_553_MOESM2_ESM.pdf]

Supplementary Figures, Tables, Notes

Supplementary Figures, Tables, Notes……………………………………………………………………………1

Supplementary Figure 1: Tools for evaluating RT-LAMP sensitivity..2

Supplementary Figure 2: Comparison of specificity in detecting of msRNA……………………………….3

Supplementary Figure 3: RT-LAMP for the precise detection of msRNA.......................................4

Supplementary Figure 4: RT-LAMP PCR curve for 120 minutes of amplification………………………..5

Supplementary Figure 5: Heatmap of the mismatches of subtype B *tat/rev* Primers binding to HIV-1 subtype B, C and A...……………………………………………………………………………………………………6

Supplementary Figure 6: Correlation of inducible reservoir size quantified by SQUHIVLA with different clinical parameters...7

Supplementary Table 1: GenBank accession numbers of the sequences used to design *tat/rev* HIV-1 msRNA specific LAMP primers and probes……………………………………………………………………8

Supplementary Table 2: *tat/rev* HIV-1 msRNA specific LAMP primers and probes sequences……9

Supplementary Table 3: *tat/rev* HIV-1 gBlock sequences…………………………………………………...10

Supplementary Table 4: RT-LAMP mastermix composition……………………………………………………11

Supplementary Table 5: Additional patient's characteristics (HIV-1 subtype B)………………………..12

Supplementary Note 1……………………………………………………………………………………………………..13

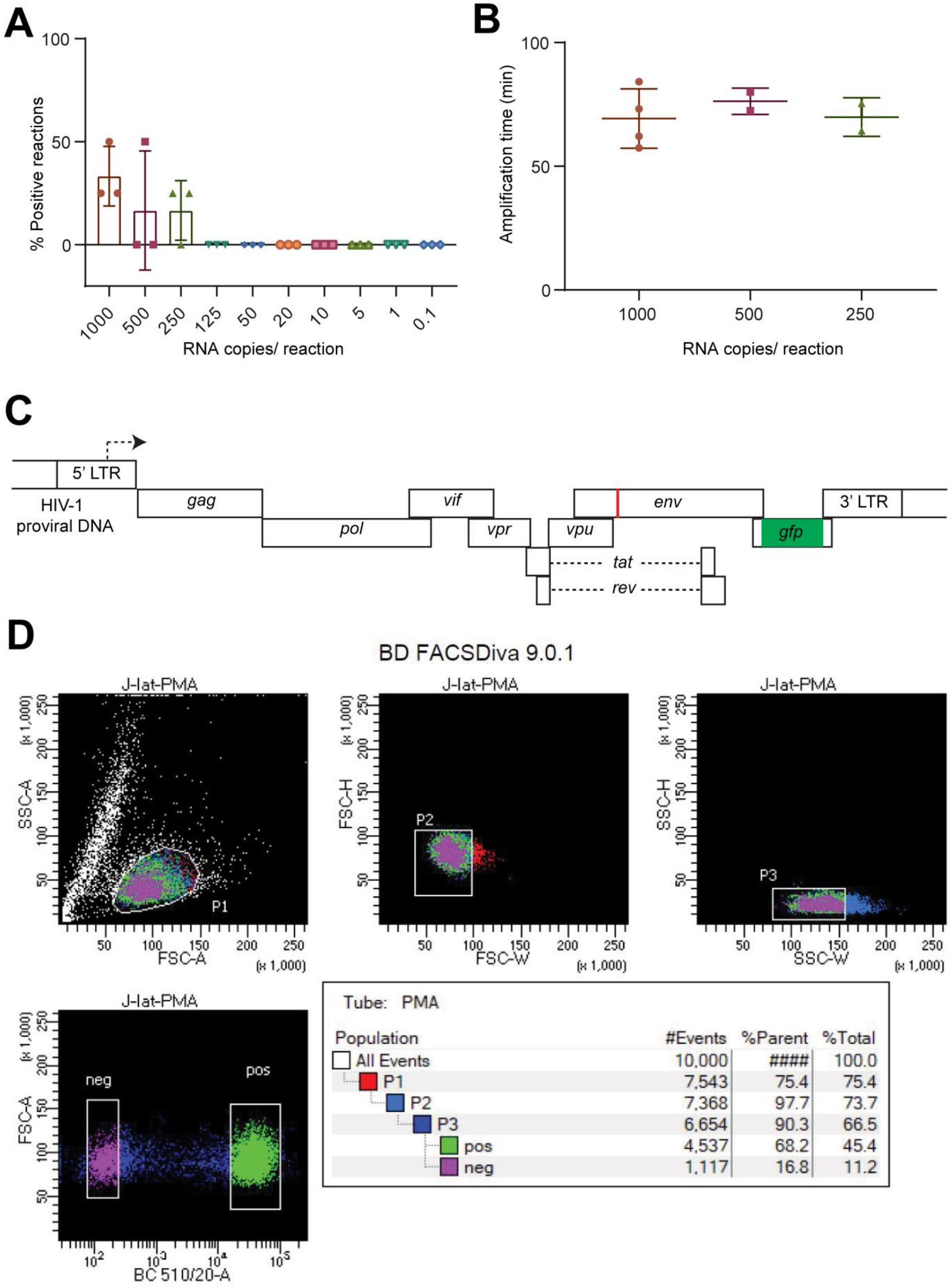
A
% Positive reactions
RNA copies/ reaction
1000 500 250 125 50 20 10 5 1 0.1
B
Amplification time (min)
RNA copies/ reaction
1000 500 250
C
5' LTR
HIV-1 proviral DNA
gag
pol
vif
vpr
vpu
tat
rev
env
gfp
3' LTR
D
BD FACSDiva 9.0.1
J-lat-PMA
SSC-A
FSC-A
P1
FSC-H
FSC-W
P2
SSC-H
SSC-W
P3
FSC-A
BC 510/20-A
neg
pos
Tube: PMA
Population #Events %Parent %Total
All Events 10,000 #### 100.0
P1 7,543 75.4 75.4
P2 7,368 97.7 73.7
P3 6,654 90.3 66.5
pos 4,537 68.2 45.4
neg 1,117 16.8 11.2

Supplementary Figure 1: Tools used for evaluating RT-LAMP sensitivity. A) Percentage of positive LAMP reactions and B) amplification time required for different amount of RNA copies are plotted when the amplification was carried out without reverse transcriptase enzyme. Data are presented as mean ± SD of three independent reactions, each of which had four technical replicates. C) Schematic overview of the integrated HIV-1 genome in JLat 11.1 cells. The green rectangle depicts the substitution of the Nef gene with GFP in the integrated HIV-1 genome, and the red rectangle represents the mutation in the HIV-1 Env gene that results in a faulty viral envelope protein. D) Sorting of GFP+ positive JLat 11.1 cells upon PMA stimulation. GFP-positive cells are represented by the green cell population, and GFP-negative cells by the purple cell population.

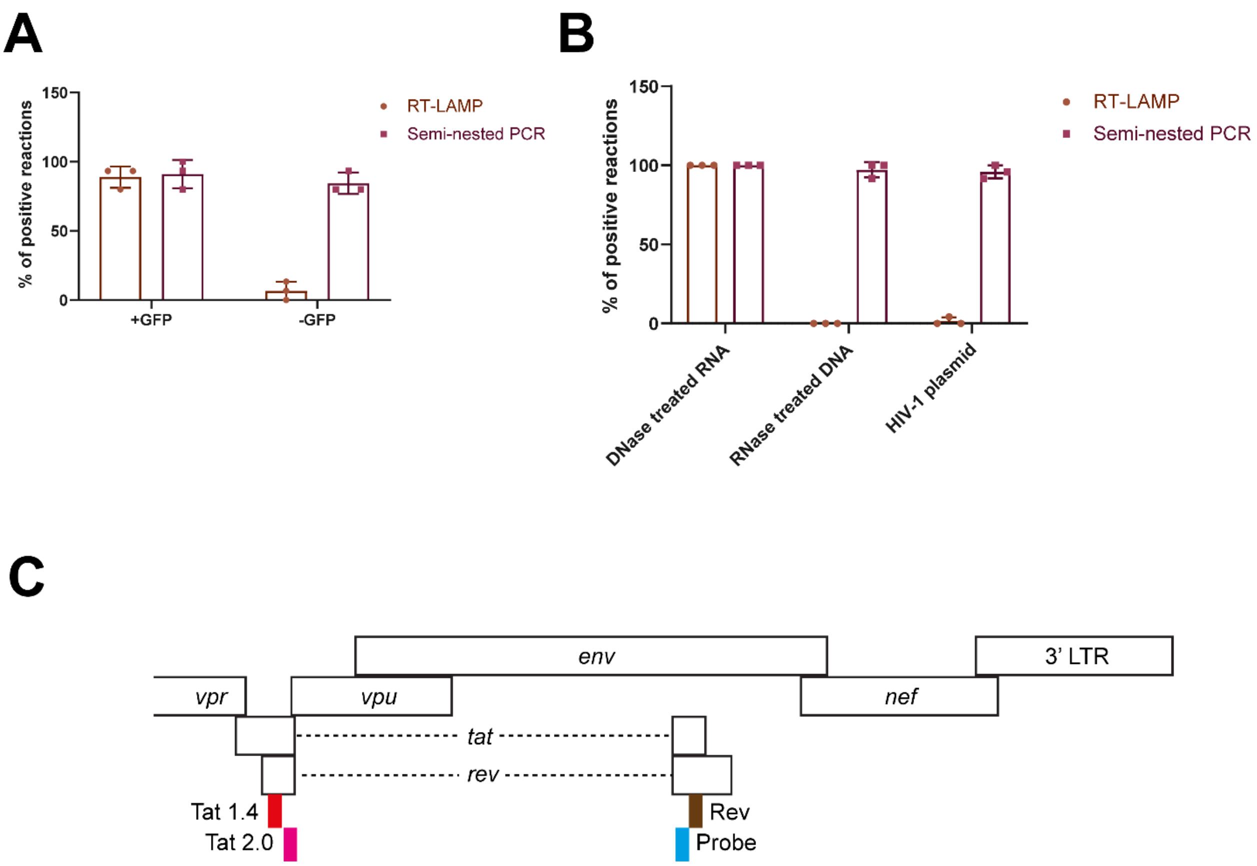

Supplementary Figure 2: Comparison of specificity in detecting of msRNA. A) Percentages of positive reactions when a single GFP+ or GFP- JLat 11.1 cell/reaction was used as input for RT-LAMP and Semi-nested PCR amplification. Data are presented as mean ± SD of three independent experiments, each of which included 15 technical replicates. B) Percentages of positive reactions when DNase treated RNA and RNase treated DNA sample isolated from PMA stimulated JLat 11.1 cells along with pNL4.3 E-R- plasmid were used as input for RT-LAMP and Semi-nested PCR

amplification. C) Schematic overview of the HIV-1 *tat/rev* specific TILDA primers and probe binding sites.

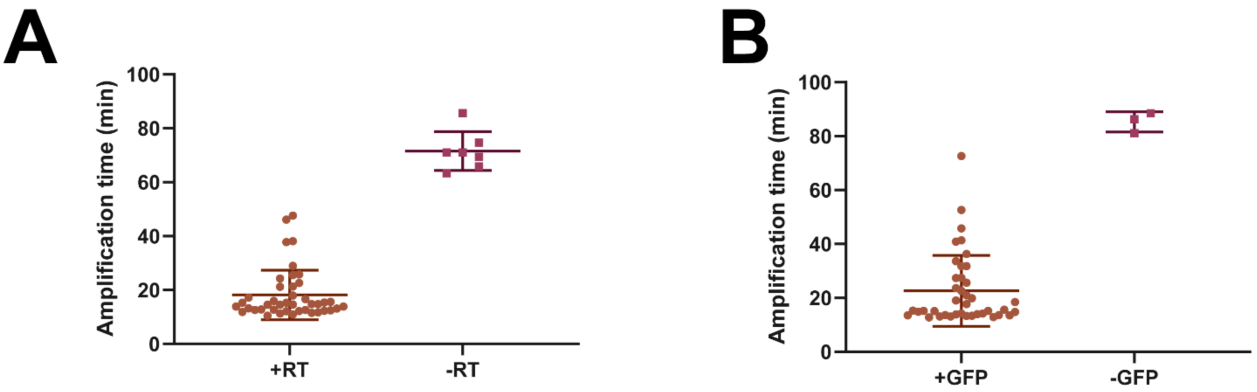

Supplementary Figure 3: RT-LAMP for the precise detection of msRNA. A) Amplification time required for RT-LAMP when a single GFP+ JLat 11.1 cell/reaction were used as input for RT-LAMP amplification with or without reverse transcriptase enzyme B) Amplification time required for RT-LAMP when a single GFP+ or GFP- JLat 11.1 cell/reaction were used as input for amplification. Data are presented as mean ± SD of three independent experiments, each of which had 15 technical replicates.

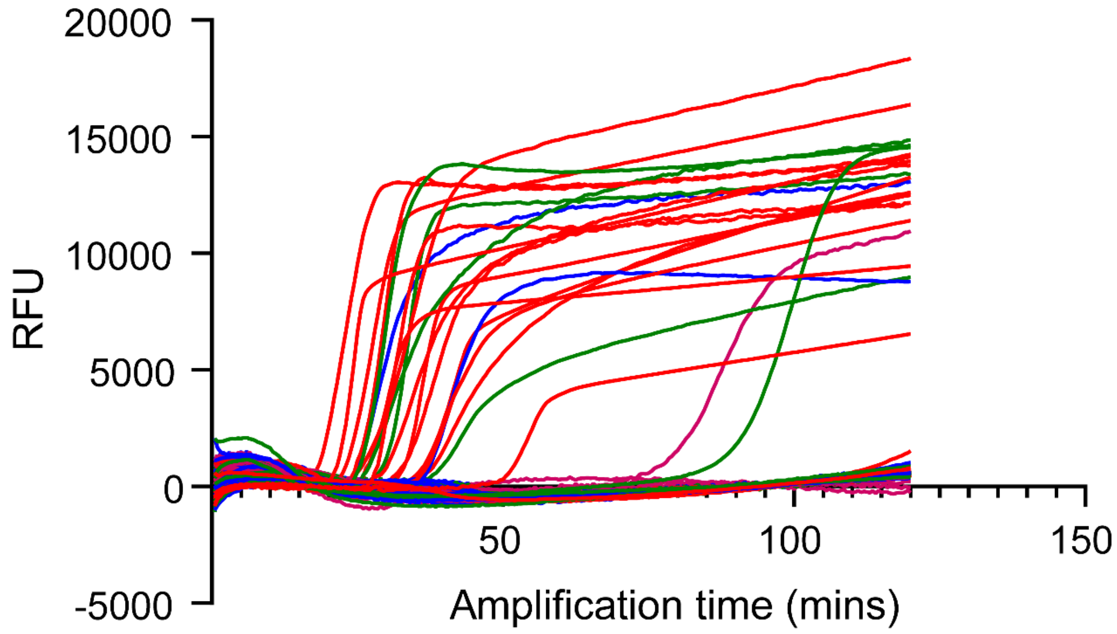

Participant ID	No. of cells/well	No. of wells	No. of positive wells	SQuHIVLa using 120 mins of amplification	SQuHIVLa using 90 mins of amplification
PLWHB1	20000 (Red)	24	14	47.71	51.97
	5000 (Green)	24	5		
	1250 (Blue)	24	2		
	313 (Magenta)	24	1		

Supplementary Figure 4: RT-LAMP PCR curve for 120 minutes of amplification.

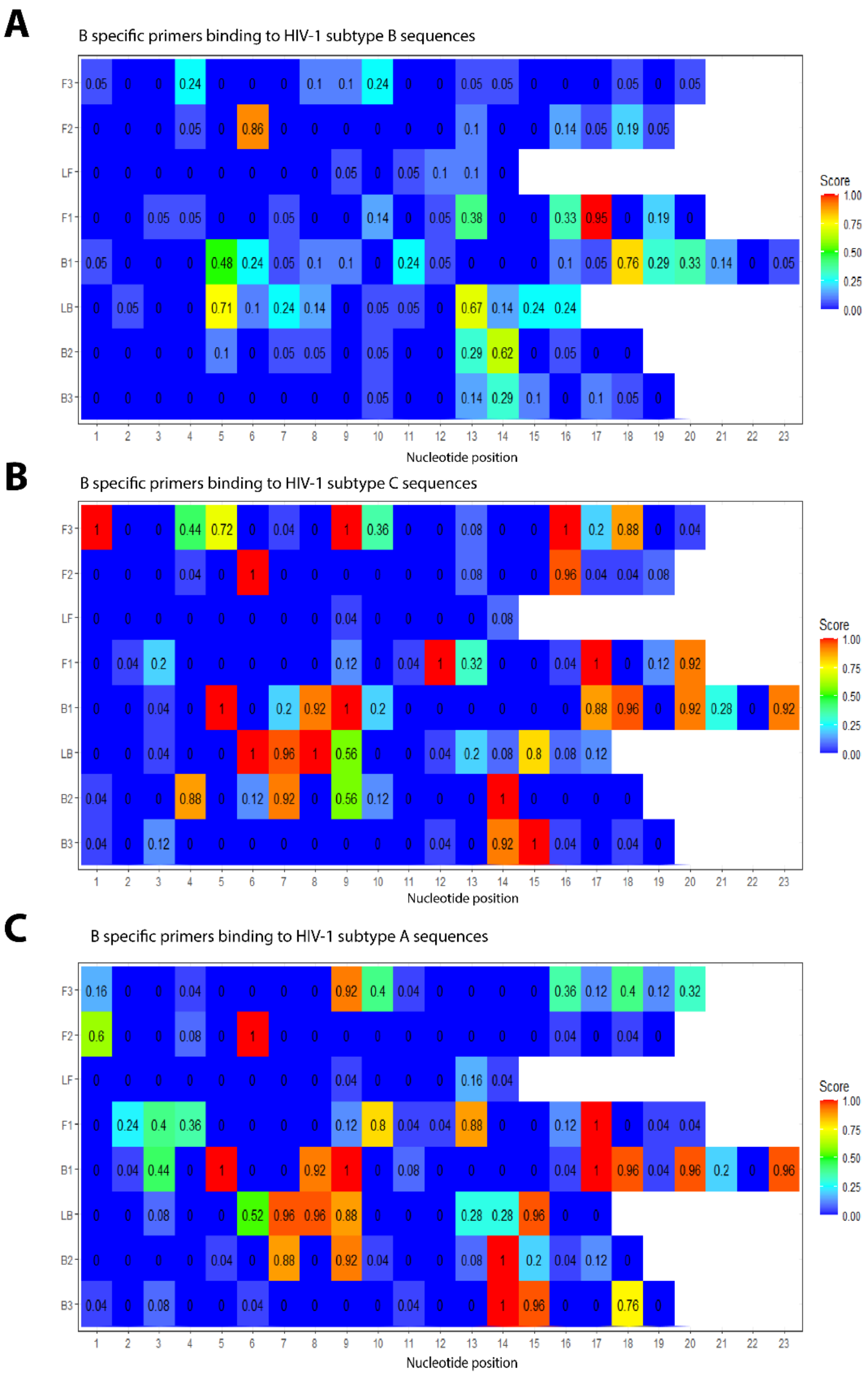

Supplementary Figure 5: Heat map of the mismatches of subtype B *tat/rev* Primers binding to HIV-1 subtype B (A), C (B) and A (C). Y-axis represents different primers and x-axis represents nucleotide positions. The value, and corresponding color, in the heat map portrays the relative amount of mismatches that occur at each position.

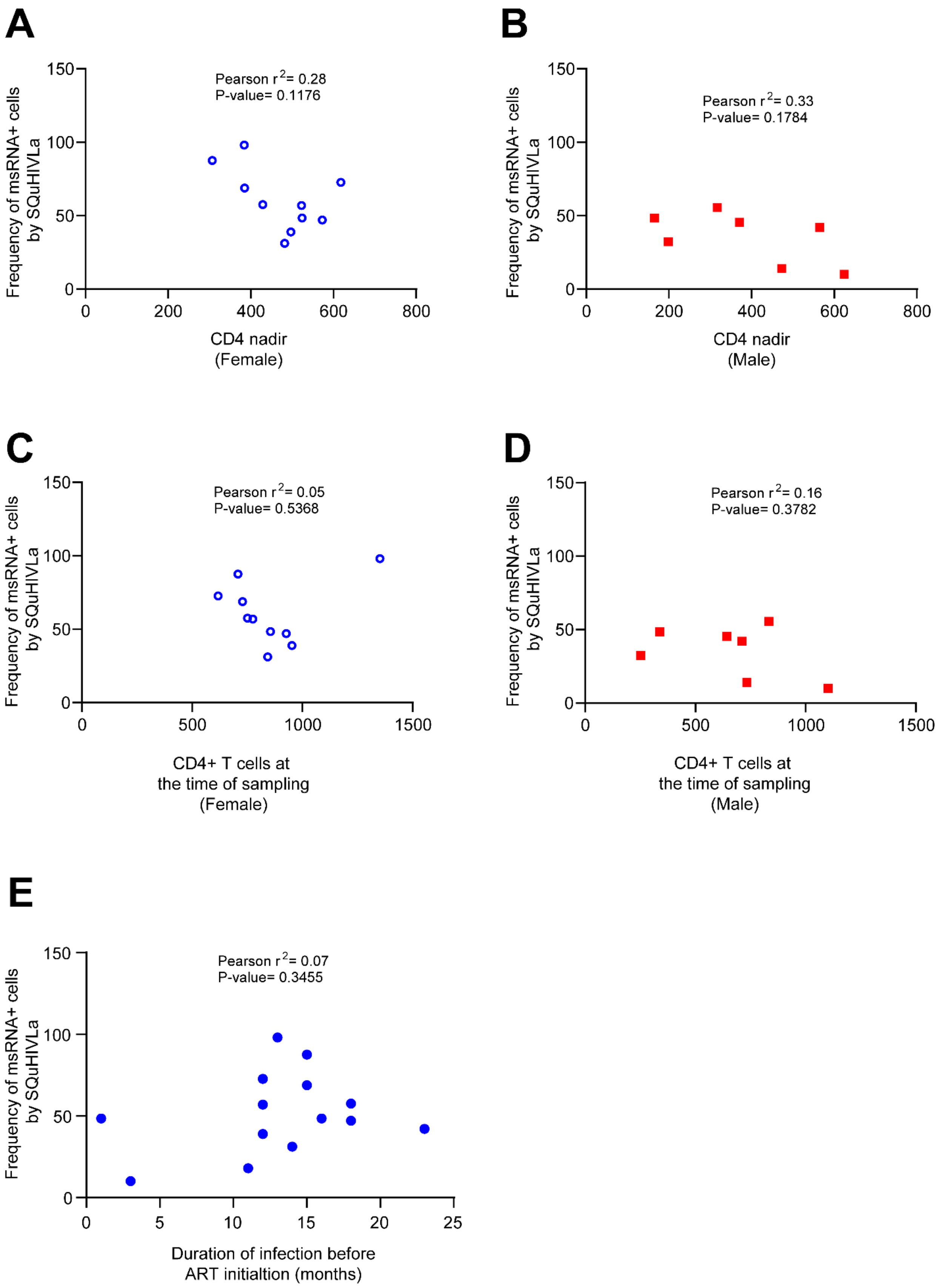
A
Pearson r2= 0.28
P-value= 0.1176
Frequency of msRNA+ cells by SQuHIVLa
CD4 nadir (Female)
B
Pearson r2= 0.33
P-value= 0.1784
Frequency of msRNA+ cells by SQuHIVLa
CD4 nadir (Male)
C
Pearson r2= 0.05
P-value= 0.5368
Frequency of msRNA+ cells by SQuHIVLa
CD4+ T cells at the time of sampling (Female)
D
Pearson r2= 0.16
P-value= 0.3782
Frequency of msRNA+ cells by SQuHIVLa
CD4+ T cells at the time of sampling (Male)
E
Pearson r2= 0.07
P-value= 0.3455
Frequency of msRNA+ cells by SQuHIVLa
Duration of infection before ART initialtion (months)

Supplementary Figure 6: Correlation of inducible reservoir size of male and female participants quantified by SQUHIVLA with different clinical parameters. The pearson correlation coefficient (r^2) is determined between inducible reservoir size quantified using SQuHIVLa and CD4 nadir for 10 female participants (A) and 7 male participants (B). The pearson correlation coefficient (r^2) is also determined between inducible reservoir size quantified using SQuHIVLa and CD4+ T cell count at the time of sampling for 10 female participants (C) and for 7 male participants (D). Female participants are depicted with blue open circle and male participants are depicted with red square. E) The pearson correlation coefficient (r^2) is determined between inducible reservoir size quantified using SQuHIVLa and duration of infection before ART initialtion for 14 participants. Statistical significance is determined by $p < 0.05$.

Supplementary Table 1: GenBank accession numbers of the sequences used to design *tat/rev* HIV-1 msRNA specific LAMP primers and probes.

**GenBank Accession numbers**	**Subtype**	**Location**	**Sampling year**
AF033819.3	B	USA	2018
A07867	B	France	1983
AB221126	B	Japan	2004
AB287364	B	Japan	2005
AB287367	B	Japan	2005
AB485638	B	USA	1991
AF042102	B	Australia	1993
AY423384	B	Netherlands	2000
AY682547	B	Russia	2004
AY779552	B	Canada	2000
AF067158	C	India	1993
AF110975	C	Botswana	1996
AY463220	C	South Africa	2000
AB254143	C	Zambia	2002
DQ369977	C	South Africa	2003
KX907339	C	Tanzania	2003
JX976688	C	South Africa	2005
MT194744	C	Zambia	2006
KU319541	C	Ethiopia	2008
KY112200	C	Malawi	2008

Supplementary Table 2: *tat/rev* HIV-1 msRNA specific LAMP primers and probes sequences

**Name**	**Sequence**
B_F3	GTGTTGCTTTCATTGCCAAG
B_B3	GTCTCTCTCTCCACCTTCT
B_FIP	TGAGGAGCTCTTCGTCGCTGCAAAAGCCTTAGGCATCTC
B_BIP	CAGTCAGACTCATCAAGTTTCTCTCTTCGATTCCTTCGGGCC
B_LF probe	TCTCCGCTTCTTCC/i6-FAMK/GC
B_LB	AACCCACCTCCCAACCC
C_F3	TCCTTGTAATAAGTGTTATTGTAA
C_B3	CGATTCTTCCGAGCCTGTC
C_FIP	CCGCTTCTTCCTGCCATAGGAATAGCTATCATTGTCTAGTTTGC
C_BIP	CGACGAAGCGCTCCTCCAAGGTTTGGGGTAAGGGTTGCT
C_LF probe	TGCCTAAGCCTTTTGTC/i6-FAMK/G
C_LB	CAGTGAGGATCATCAAAATC

Supplementary Table 3: *tat/rev* HIV-1 gBlock sequences

**Name**	**Sequence**
HIV-1 subtype b gBlock	TAATACGACTCACTATAGGGGCGGCCGCATGGAGCCAGTAGATCCTAGACTAGAGCC<br>CTGGAAGCATCCAGGAAGTCAGCCTAAGACTGCTTGTACCAATTGCTATTGTAAAAAG<br>TGTTGCTTTCATTGCCAAGTTTGTTTCATAACAAAAGCCTTAGGCATCTCCTATGGCAG<br>GAAGAAGCGGAGACAGCGACGAAGAGCTCCTCAGGACAGTCAGACTCATCAAGTTTC<br>TCTATCAAAGCAACCCACCTCCCAGCCCCGAGGGGACCCGACAGGCCCGAAGGAATC<br>GAAGAAGAAGGTGGAGAGAGAGACAGAGACAGATCCGGTCGATTAGTGAATGGATT<br>CTTAGCACTTATCTGGGACGACCTGCGGAGCCTGTGCCTCTTCAGCTACCACCGCTTGA<br>GAGACTTACTCTTGATTGTAACGAGGATTGTGGAACTTCTGGGACGCAGGGGGTGGG<br>AAGCCCTCAAATATTGGTGGAATCTCCTACAGTATTGGAGTCAGGAACTAAAGAATAG<br>CCTCGAGG
HIV-1 subtype c gBlock	TAATACGACTCACTATAGGGGCGGCCGCATGGAGCCAGTAGATCCTAACCTAGAGCCC<br>TGGAACCATCCAGGAAGTCAGCCTAAAACTCCTTGTAATAAGATGTTATTGTAAAAAAT<br>GTAGCTATCATTGTCTAGTTTGCTTTCAGACAAAAGGCTTAGGCATTTCCTATGGCAGG<br>AAGAAGCGGAGACAGCGACGAAGCGCTCCTCCAAGCAGTGAGGATCATCAAAATCTT<br>ATATCAAAGCAACCCTTACCCCAAACCCGAGGGGACCCGACAGGCTCGGAAGAATCG<br>AAGAAGAAGGTGGAGAGCAAGACAGAGACAGATCCATTCGATTAGTGAACGGATTCT<br>TAGCACTTGCCTGGGACGACCTGCGGAGCCTGTGCCTTTTCAGCTACCACCGATTGAG<br>AGACTTCATATTGGTTGCAGCGAGAGCGGTGGAACTTCTGGGACGCAGCAGTCTCAG<br>GGGACTACAGAGGGGGTGGGAAGCCCTTAACCTCGAGG

Brown = T7 promoter sequence

Green = HIV-1 *tat/rev* consensus sequence of the mentioned subtype

Supplementary Table 4: RT-LAMP mastermix composition.

**Components and stock conc.**	**Volume used**	**Final Conc.**
10X Isothermal Amplification Buffer	2 µL	1X
MgSO4 (100 mM)	1.2 µL	6 mM (8 mM total)
dNTP Mix (10 mM)	2.8 µL	1.4 mM
FIP/BIP Primers (32 µM)	1 µL	1.6 µM
F3/B3 Primers (8 µM)	0.5 µL	0.2 µM
LF probe/LB Primer (16 µM)	0.5 µL	0.4 µM
Bst 2.0 WarmStart DNA Polymerase (8000 units/mL)	1 µL	8 units
WarmStart RTx Reverse Transcriptase (15000 units/mL)	0.5 µL	7 units
RNasin® RNase Inhibitor (40 units/ µL)	0.5 µL	20 units
Triton 4%	5 µL	1%
Template	5 µL	
**Total Reaction Volume**	**20 µL**	

Supplementary Table 5: Additional patient's characteristics (HIV-1 subtype B).

Patient ID	*Age (years)*	*Sex assigned at birth*	*ART regimen*	*Viral load at sample collection*	*CD4+ T cell count (cells/mm^3)*	*Duration of viral suppression <50 c/mL (years)*	*CD4 nadir (cells/mm^3)*	*Pre-cART plasma HIV-1 RNA (log10 copies/mL)*	Inducible reservoir by SQuHIVLa (cells/millions of CD4+ T cells
PLWHB1	56-60	M	TAF, FTC, RPV	<30	470	11-15	290	65900	51.97
PLWHB2	71-75	M	TAF, FTC, NVP	<30	800	11-15	250	24000	133.1
PLWHB3	61-65	M	ABC, 3TC, DTG	<30	780	6-10	290	104000	106.2
PLWHB01	41-45	M	TDF, 3TC, NVP	<50	570	1-5	290	205000	33.14
PLWHB02	55-60	M	3TC, AZT, NVP	<50	590	1-5	250	53300	65.11
PLWHB03	45-50	M	3TC, AZT, NVP	<50	940	1-5	70	102000	40.62
PLWHB04	41-45	M	TDF, 3TC, NVP	<50	510	1-5	190	107000	27.00
PLWHB05	41-45	M	ABC, 3TC, NVP	<50	580	1-5	150	102000	36.78
PLWHB06	36-40	M	FTC, TDF, NVP	<50	680	1-5	160	70000	39.06
PLWHB07	46-50	M	3TC, TDF, ATV/r	<50	650	1-5	240	142000	21.75
PLWHB08	31-35	M	FTC, TAF, NVP	<20	810	6-10	300	218000	52.64
PLWHB09	51-55	M	3TC, DTG	<20	650	6-10	240	72500	35.70
PLWHB10	56-60	M	FTC, TDF, NVP	<20	400	16-20	150	75300	82.44
PLWHB11	51-55	M	FTC, TAF, DTG	<20	610	6-10	320	79000	14.27
PLWHB12	46-50	M	3TC, ABC, DTG	<20	470	10-15	200	75700	28.76

Supplementary Note 1:

Steps to design HIV-1 *tat/rev* msRNA LAMP primers:

1) Select reference HIV-1 genome sequences

To design LAMP primers for a specific subtype of HIV-1, download a minimum of 10 complete genome sequences of the desired subtype from the Los Alamos database using the sequence search interface webpage (https://www.hiv.lanl.gov/components/sequence/HIV/search/search.html). Select sequences submitted from different geographic locations (such as, Africa, Asia, Europe and USA) and in different years (e.g., sequences submitted before 2000, between 2000-2010 and 2010-present). This selection is necessary to account for sequence diversity and increase the probability that the designed primers will recognize and bind to *tat/rev* msRNA from a majority of individuals with the selected HIV-1 subtype. Download the selected reference HIV-1 genome sequences, save in FASTA file format.

2) Generate a spliced *tat/rev* consensus sequence *in silico*

Align the downloaded reference HIV-1 genome sequences using a sequence alignment tool of choice. We utilized the built-in MUSCLE algorithm in MEGA11 software. Manually edit the aligned sequences by excluding the following sequence regions; upstream of the Tat gene (1 to 5830); *tat/rev* intron (6046 to 8383) and downstream of the *rev* gene (8654 to 3'end). The nucleotide positions are relative to the coordinates of the 5' LTR UR start of the reference HIV-1 genome (ID) provided in the Los Alamos HIV-1 sequence compendium (2021). The resulting alignment of the spliced *tat/rev* sub-genomic region should be approximately 490 nucleotides. Download the spliced *tat/rev* DNA consensus sequence, save in FASTA file format.

Generate a preliminary LAMP primer set using the PrimerExplorer V5 software

Upload the *tat/rev* DNA consensus FASTA file (i.e., the target sequence file) into the online PrimerExplorer V5 software. Adjust the default settings prior to executing the primer design algorithm. Set the sequence parameter condition to "AT rich"; the GC content of the *tat/rev* sequence varies from 35-45% depending on the HIV-1 subtype. Set the Tm for F1c/B1c to 60°C-

64°C, and set the Tm for F3/B3 and F2/B2 to 55°C-60°C to ensure the generation of a sufficient number of potential LAMP primer sets. The software displays a maximum of 1000 primer sets using a combination of different primer binding sites.

Several prerequisites need to be considered when selecting the preliminary primer set from the potential LAMP primer sets generated by the software.

A) To ensure the specificity of the primer set for *tat/rev* msRNA and not the intron-containing *tat/rev* DNA, only primer sets containing the splice site of msRNA (approximately, the 215th nucleotide) within the F2 to F1c or B2 to B1c primer binding regions should be considered. This ensures that unstable loop formation, while the primers are bound to intron-containing *tat/rev* DNA, would result in the inhibition of isothermal amplification. Therefore, select potential primer sets where the F2 binding region is within 25 nucleotides downstream of the splice site and 90 nucleotides upstream of the splice site (approximately, nt pos. 125 to 240) or where the B2 binding region is within 25 nucleotides upstream of the splice site and 90 nucleotides downstream of the splice site (approximately, nt pos. 190 to 305).

B) Since loop primers, which accelerate the LAMP reaction, are also used for amplification, only primer sets that have a distance of >22 nucleotides between F2 and F1c/B2 and B1c should be considered for the preliminary primer set.

C) If impossible to find one primer set that fulfills both requirements for exclusive binding of msRNA, and suitable for designing the loop primers, then primer binding sites belonging to different primer sets could be mixed to form a custom set as long as they have a similar Tm for F3/B3, F2/B2, and F1c/B1c and fulfill the specific distance requirement between LAMP primer binding regions.

After selecting the preliminary primer set, download the sequences and primer information files from the software to use for designing the Loop primers (LF/LB).

3) Design loop primers

Upload the LAMP primer information files into the PrimerExplorer V5 software. Since one of the loop primers needs to be converted into a self-quenching and internal FAM fluorophore-containing probe, specific criteria should be considered when selecting the loop primers:

A) presence of a cytosine (C) or guanine (G) residue at the terminal 3′ end
B) a thymine (T) residue at the second or third position from this 3′ end
C) one or more G nucleotides flanking the T residue (optional).

In case no suitable loop primers can be selected from the software-generated list, the closest possible sequence is chosen, and manual modifications are performed to ensure its applicability as a probe (detailed below).

4) Adapt LAMP primer and probe sequences

The *tat/rev* region of HIV-1 is highly diverse, making it difficult to find completely conserved primer binding regions for LAMP primers. To address this, align the primer binding regions of the preliminary primer set selected in **step 3** to the reference HIV-1 sequences downloaded from the Los Alamos database in **step 1**. Identify mutation hotspots located within primer binding regions that would result in primer-template mismatches. Ensure that the primers contain conserved nucleotides at specific positions that are essential for amplification to proceed (3′ end of F2/B2 and F3/B3, and 5′ end of F1c/B1c. Mutation hotspots are permitted at less essential regions (5′ end of F2/B2 and F3/B3, 5′ end of the F1c/B1c, and the internal region) where a mismatch will have less effect on amplification. To ensure these criteria, shift the preliminary primers upstream or downstream of the current primer binding site. Recalculate the Tm for each modified primer to ensure that the new primers have similar Tm to the original primers, and that the distance between the modified primer regions must also meet LAMP-specific requirements. The distances between the primer binding regions are calculated from the alignment and the Tm of the modified primers is calculated using Kun's Oligonucleotide Tm calculator (https://arep.med.harvard.edu/kzhang/cgi-bin/myOligoTm.cgi). The calculated Tm is affected by experimental conditions such as the salt concentration and oligo concentration, so it is preferred that Tm be calculated under fixed experimental conditions (oligo concentration at 0.1 µM, sodium ion concentration at 50 mM, magnesium ion concentration at 4 mM).

Similarly, align the selected loop primers to the reference HIV-1 complete genome sequences and modify following the same procedures (shifting the loop primers upstream or downstream of the current primer binding site). Ensure that one of the loop primers fulfills the requirements to be converted into a self-quenching probe and that the internally labeled T and 3’ end G/C of the probe region are conserved among the HIV-1 sequences used for the alignment.

The sequences of the HIV-1 genotype B and C LAMP primers and probes designed and used to validate specific detection of *tat/rev* msRNA by RT-LAMP are provided in Supplementary Table 2.
