# Supplementary material for: Specific quantification of inducible HIV-1 reservoir by RT-LAMP
Source: Commun Med (Lond). 2024 Jun 25;4:123. doi: 10.1038/s43856-024-00553-4 (PMC11199587; doi:10.1038/s43856-024-00553-4)
Supplement: Supplementary file 3 — Description of Additional Supplementary Files [file 43856_2024_553_MOESM3_ESM.pdf]

Description of Additional Supplementary Files

File name: Supplementary data 1.

File Description: Source Data.
