# Supplementary material for: Specific quantification of inducible HIV-1 reservoir by RT-LAMP
Source: Commun Med (Lond). 2024 Jun 25;4:123. doi: 10.1038/s43856-024-00553-4 (PMC11199587; doi:10.1038/s43856-024-00553-4)
Supplement: Supplementary file 5 — Reporting Summary [file 43856_2024_553_MOESM5_ESM.pdf]

nature portfolio

Corresponding author(s): Tokameh Mahmoudi

Last updated by author(s): Mar 21, 2024

Reporting Summary

Nature Portfolio wishes to improve the reproducibility of the work that we publish. This form provides structure for consistency and transparency in reporting. For further information on Nature Portfolio policies, see our Editorial Policies and the Editorial Policy Checklist.

Statistics

For all statistical analyses, confirm that the following items are present in the figure legend, table legend, main text, or Methods section.

n/a	Confirmed	
☐	☒	The exact sample size (*n*) for each experimental group/condition, given as a discrete number and unit of measurement
☐	☒	A statement on whether measurements were taken from distinct samples or whether the same sample was measured repeatedly
☐	☒	The statistical test(s) used AND whether they are one- or two-sided<br>*Only common tests should be described solely by name; describe more complex techniques in the Methods section.*
☒	☐	A description of all covariates tested
☐	☒	A description of any assumptions or corrections, such as tests of normality and adjustment for multiple comparisons
☐	☒	A full description of the statistical parameters including central tendency (e.g. means) or other basic estimates (e.g. regression coefficient) AND variation (e.g. standard deviation) or associated estimates of uncertainty (e.g. confidence intervals)
☒	☐	For null hypothesis testing, the test statistic (e.g. *F*, *t*, *r*) with confidence intervals, effect sizes, degrees of freedom and *P* value noted<br>*Give P values as exact values whenever suitable.*
☒	☐	For Bayesian analysis, information on the choice of priors and Markov chain Monte Carlo settings
☒	☐	For hierarchical and complex designs, identification of the appropriate level for tests and full reporting of outcomes
☐	☒	Estimates of effect sizes (e.g. Cohen's *d*, Pearson's *r*), indicating how they were calculated

Our web collection on statistics for biologists contains articles on many of the points above.

Software and code

Policy information about availability of computer code

Data collection	For RT-LAMP and RT-qPCR, experiments were performed on CFX96 Touch Real-Time System Thermocycler (BioRad) using CFX Maestro software version 2.2.
Data analysis	For RT-LAMP and RT-qPCR, date were analysed using CFX Maestro version 2.2. Lamp primers were designed with the help of PrimerExplorer V5 software. All graphs were generated and graphical abstract were performed using GraphPad Prism version 8.0.2 for Windows. All the schematics were prepared using BioRender.com.

For manuscripts utilizing custom algorithms or software that are central to the research but not yet described in published literature, software must be made available to editors and reviewers. We strongly encourage code deposition in a community repository (e.g. GitHub). See the Nature Portfolio guidelines for submitting code & software for further information.

Data

Policy information about availability of data

All manuscripts must include a data availability statement. This statement should provide the following information, where applicable:

- Accession codes, unique identifiers, or web links for publicly available datasets
- A description of any restrictions on data availability
- For clinical datasets or third party data, please ensure that the statement adheres to our policy

All data needed to evaluate the conclusions in the paper are present in the paper and/or the Supplementary information. All source data used in the manuscript is provided as a Supplementary data 1. Additional data related to this paper may be requested from the authors.

Research involving human participants, their data, or biological material

Policy information about studies with human participants or human data. See also policy information about sex, gender (identity/presentation), and sexual orientation and race, ethnicity and racism.

Reporting on sex and gender	Information about biological sex assigned at birth for all the participants were included in the Table 1 and Supplementary table 5.
Reporting on race, ethnicity, or other socially relevant groupings	Not applicable.
Population characteristics	HIV-1 infected donors used in this study were older than 18 years, on fully suppressive cART, with non detectable viral loads for more than 12 months. The population characteristics of human research participants are described in Table 1 and supplementary table 5 of the manuscript.
Recruitment	HIV-1 infected donors were recruited based on voluntary enrollment to participate in the study and provided informed, signed consent. There are no self-selection or other biases that can influence results.
Ethics oversight	he study protocol was approved by the Medical Ethical Council of the Erasmus Medical Center (MEC-2012–583) and University of KwaZulu-Natal (REF: E036/06).

Note that full information on the approval of the study protocol must also be provided in the manuscript.

Field-specific reporting

Please select the one below that is the best fit for your research. If you are not sure, read the appropriate sections before making your selection.

☒ Life sciences ☐ Behavioural & social sciences ☐ Ecological, evolutionary & environmental sciences

For a reference copy of the document with all sections, see nature.com/documents/nr-reporting-summary-flat.pdf

Life sciences study design

All studies must disclose on these points even when the disclosure is negative.

Sample size	Experiments done with cell line J-Lats 11.1 were performed at least three times, or as indicated in the figure legends. For experiments using primary CD4+ T cells obtained from human donors, sample sizes were indicated in the figure legends. The sample size was determined based on the minimum requirement to perform statistical tests and the availability of materials.
Data exclusions	No data was excluded in these studies.
Replication	All attempts at replication of this data were successful. All experiments had at least three independent replicates and / or technical replicates as indicated in the figure legends.
Randomization	Samples were independently and randomly analyze.
Blinding	Samples were blindly analyzed.

Reporting for specific materials, systems and methods

We require information from authors about some types of materials, experimental systems and methods used in many studies. Here, indicate whether each material, system or method listed is relevant to your study. If you are not sure if a list item applies to your research, read the appropriate section before selecting a response.

Materials & experimental systems

n/a	Involved in the study
☒	☐ Antibodies
☐	☒ Eukaryotic cell lines
☒	☐ Palaeontology and archaeology
☒	☐ Animals and other organisms
☒	☐ Clinical data
☒	☐ Dual use research of concern
☒	☐ Plants

Methods

n/a	Involved in the study
☒	☐ ChIP-seq
☒	☐ Flow cytometry
☒	☐ MRI-based neuroimaging

Eukaryotic cell lines

Policy information about cell lines and Sex and Gender in Research

Cell line source(s)	J-Lat 11.1 cell line was obtained from the Verdin and Greene Laboratories at the Gladstone Institutes, which generated these lines from Jurkat cells.
Authentication	J-Lat 11.1 cells were authenticated by the Verdin and Greene Laboratories at the Gladstone Institutes using PCR assays with species-specific primers.
Mycoplasma contamination	All cell lines were tested negative for mycoplasma testing.
Commonly misidentified lines (See ICLAC register)	No commonly misidentified cell lines were used in this study.

Plants

Seed stocks	Not applicable.
Novel plant genotypes	Not applicable.
Authentication	Not applicable.
